# The impact of phthalates on asthma and chronic obstructive pulmonary disease: a comprehensive analysis based on network toxicology and molecular docking

**DOI:** 10.3389/fphar.2025.1566965

**Published:** 2025-03-14

**Authors:** Ren Li, Bingqing Zheng, Yuqiong Zhang, Lu He, Chaomin Ren, Linlin Guan, Huan Yang, Jiayu Tian, Xingyi Chen, Dongxing Shi, Lifang Zhao, Zhihong Zhang

**Affiliations:** ^1^ Department of Environmental Health, School of Public Health, Shanxi Medical University, Taiyuan, China; ^2^ Center for Ecological Public Health Security of Yellow River Basin, Shanxi Medical University, Taiyuan, China; ^3^ Key Laboratory of Coal Environmental Pathogenicity and Prevention (Shanxi Medical University), Ministry of Education, Taiyuan, China; ^4^ Department of Public Health Laboratory Sciences, School of Public Health, Shanxi Medical University, Taiyuan, China

**Keywords:** phthalates, asthma, COPD, network toxicology, molecular docking

## Abstract

**Introduction:**

Phthalates (PAEs) are widely used plasticizers in polyvinyl chloride (PVC) products since the 1930s, and recent research indicates a significant association between exposure to these substances and the development and progression of asthma and chronic obstructive pulmonary disease (COPD). Understanding the underlying mechanisms is crucial due to their public health implications.

**Methods:**

In this study, we utilized innovative network toxicology and molecular docking techniques to systematically examine the effects of seven typical phthalates on asthma and COPD. By integrating information from multiple databases, we identified key target genes linked to these compounds and conducted functional enrichment analyses to elucidate their roles in pathological processes.

**Results:**

Our results demonstrate that these phthalates affect the pathogenesis of asthma and COPD by modulating various target genes, including PTGS2, MMP9, and CASP3, which are involved in essential biological pathways such as apoptosis and immune response. Interestingly, certain inflammation-related genes and signaling pathways displayed novel regulatory patterns when exposed to phthalates, revealing new pathological mechanisms. Molecular docking analyses further confirmed stable interactions between phthalates and essential target genes, providing molecular-level insights into their pathogenic mechanisms.

**Discussion:**

Overall, this study highlights the harmful impacts of these seven phthalates on asthma and COPD, establishing new connections between the compounds and disease-related genes, and emphasizing their relevance as emerging environmental toxins. These findings provide valuable perspectives for risk assessment and public health policy, underscoring the need for stricter regulations and interventions regarding phthalate exposure.

## 1 Introduction

Di-phthalates, also known as phthalates (PAEs), are synthetic chemicals that have been utilized as plasticizers in polyvinyl chloride (PVC) products since the 1930s. PAEs are classified into two categories according to their molecular weight: low molecular weight phthalates (LMW) and high molecular weight phthalates (HMW). LMW phthalates, including dimethyl phthalate (DMP), diethyl phthalate (DEP), diisobutyl phthalate (DiBP), and di-n-butyl phthalate (DBP), are primarily used in decorative materials such as paints, varnishes, tiles, sealants, and welds. HMW phthalates, such as di (2-ethylhexyl) phthalate (DEHP), diisononyl phthalate (DiNP), and dioctyl phthalate (DOP), are primarily used in applications like construction, building materials, toys, and food packaging (Arbuckle et al., 2014; Kasper-Sonnenberg et al., 2014; Marie et al., 2015). In this paper, the above seven phthalates were selected for study. As phthalate plasticizers are physical, not chemically, bound to the polymer system, even minor environmental changes—such as variations in pH, temperature, pressure, exposure to irradiation, or contact with lipids—can increase the leaching, migration, or evaporation of phthalates from the plastic material into the surrounding environment, thereby presenting a potential risk to human health (Benjamin et al., 2015). The industrial uses, environmental behavior, and primary human exposure pathways of phthalates are determined by their physical and chemical properties. Human exposure to PAEs occurs through three main routes: inhalation, ingestion, and dermal absorption (Zhao et al., 2022). Various studies highlight the potential harmful effects of different exposure pathways on both human and animal health (Zhao et al., 2022; Brassea-Pérez et al., 2022).

Evidence linking phthalate exposure to increased risk of respiratory diseases (Berger et al., 2020). Phthalates have been associated with asthma, airway inflammation, and reduced lung function in an increasing number of epidemiological studies, with early studies of phthalate exposure and respiratory disease dating back to the 1970s (Polakoff et al., 1975). Asthma is identified as the most common chronic respiratory disease worldwide in the Global Burden of Disease study (GBD 2015 Chronic Respiratory Disease Collaborators, 2017), its main features include chronic airway inflammation, airway hyperresponsiveness, and airway remodeling. In recent years, the prevalence of asthma has gradually increased, and the number of asthma sufferers worldwide has reached 358 million, causing enormous health pressure and economic burden (Liye et al., 2024). Chronic obstructive pulmonary disease (COPD) is a chronic respiratory disease that shares certain characteristics with asthma. Both conditions are characterized by airway obstruction and chronic inflammation (Barnes, 2008). The pathological types are similar, often in the form of co-morbidities, and persistent airflow limitation leading to pulmonary ventilation dysfunction is a characteristic pathological change in COPD. A review of the literature reveals that the prevalence of COPD in adults over the age of 40 is estimated to be between 9 and 10 percent (Halbert et al., 2006). As indicated by data from the World Health Organization (WHO) in 2019, COPD represents the third leading cause of mortality on a global scale.

Increasing prevalence of Asthma and COPD likely influenced by environmental risk factors (Leynaert et al., 2019; Hulin et al., 2012). The relationship between phthalate exposure and respiratory diseases, particularly asthma, has been the subject of growing interest in recent years (Lee et al., 2020). Epidemiological data indicate a potential correlation between phthalate exposure and the development of childhood asthma and airway disease (Jurewicz and Hanke, 2011; Adgent et al., 2020; Odebeatu et al., 2019). The findings of a recent study indicate a correlation between urinary phthalate metabolites and the onset of asthma, thereby reinforcing the hypothesis that phthalate exposure may be a contributing factor in the development of this respiratory condition (Duh et al., 2023). In a separate study, exposure to specific phthalates (DEHP and DBP) was linked to an increased prevalence of COPD among a subset of COPD patients (Quirós-Alcalá et al., 2023). Based on various epidemiologic evidence, a safety assessment of phthalates for respiratory diseases is necessary.

To comprehensively examine the deleterious effects of seven phthalates on asthma and COPD, we employed network toxicology and molecular docking techniques. Network toxicology is the interdisciplinary integration of different scientific fields, including bioinformatics, big data analysis, genomics, and other related technologies (Tao et al., 2013). The objective is to construct a network of relationships between compounds, toxicity, and targets by utilizing multiple databases (Huang, 2024). It is recommended that the interactions and toxic pathways be visualized in order to provide a more comprehensive explanation of the toxic effects. In contrast to traditional toxicology, network toxicology has transformed the “one-target-one-drug” model into a novel paradigm of “multi-target-multi-drugs” (Li, 2016). Molecular docking is a computational technique that simulates complex binding patterns to protein targets, thereby elucidating potential mechanistic pathways that may be involved in the pathogenesis of asthma and COPD (He et al., 2024). There are many studies confirming that molecular docking technology can be used in the development of vaccines for cancer treatment (Nainwal et al., 2022; Nainwal et al., 2020; Nainwal et al., 2014) and infectious diseases (Nabi et al., 2022) with better results. There have also been studies utilizing molecular docking to find therapeutic interventional agents for respiratory systems such as COPD (Arora et al., 2022a). The objective of this study is to contribute to the advancement of knowledge regarding the treatment and prevention of respiratory diseases by employing advanced methodologies to elucidate the molecular mechanisms underlying the effects of phthalates on asthma and COPD.

## 2 Materials and methods

**FIGURE 1:**
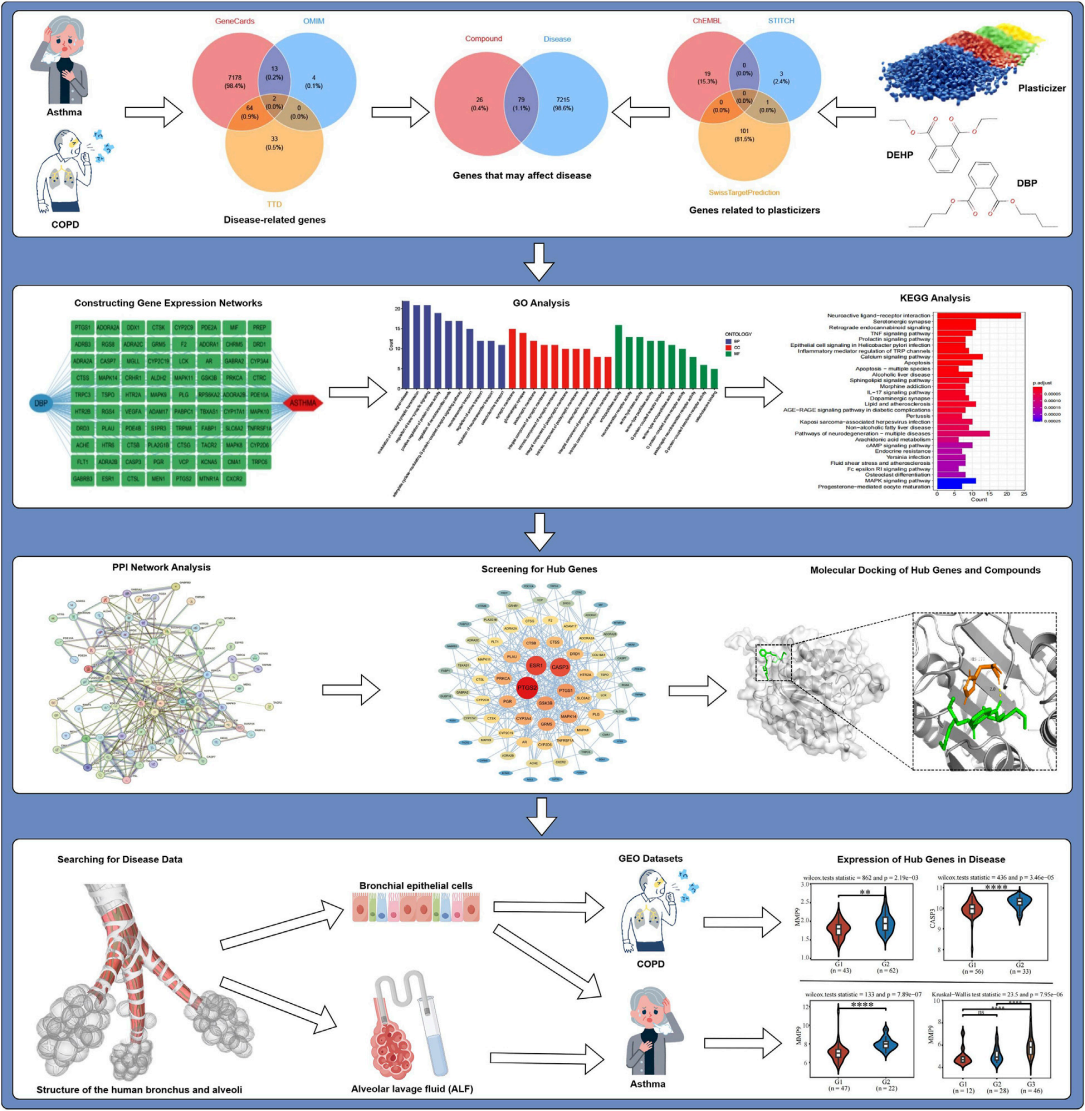
Flowchart for the analysis of this study.

All the databases and access links used in this paper have been placed in Supplementary Table S1. Figure 1 shows the flowchart of the research program.

### 2.1 Retrieval of chemical structure of plasticizers

The seven most common chemical constituents of plasticizers were identified through a comprehensive search of PubMed, Google Scholar, and other relevant databases.

### 2.2 Toxicity studies of plasticizers

Literature search for plasticizer-related respiratory diseases, especially asthma and COPD, using databases such as PubMed, Google Scholar, etc.

### 2.3 Acquisition of chemical structure

The SMILES structures of the seven main plasticizer components were searched in the PubChem database. Subsequently, the targets of these components were searched in other databases, including ChEMBL, STITCH, and SwissTargetPrediction. The target genes of these compounds were then extracted and assembled.

### 2.4 Acquisition of disease-related target genes

Download the relevant target genes in Genecards (screening genes is conditional on scoring more than 10 points), OMIM, and TTD databases. To take the concatenation set of these disease-related target genes. And take the intersection set with the previously obtained compound target genes to get the set of genes whose compounds may affect the disease. From there, subsequent analysis is performed.

### 2.5 Construction and enrichment analysis of protein interaction networks

The STRING database was employed to construct the relationship network of target genes for the major components of asthma, COPD, and plasticizers (confidence level set at 0.4). We then performed GO enrichment analysis to elucidate biological functions and KEGG enrichment analysis to identify biological pathways (all results were FDR-corrected, and a corrected *P* < 0.05 was considered significant for the analysis).

### 2.6 Core hub gene screening

We entered the intersecting genes representing potential targets for COPD and asthma pathogenesis into the STRING database, respectively. The confidence level was set to a medium confidence level of 0.4, and the interactions network data were acquired for subsequent screening of pivotal genes, as well as for PPI network interactions maps (in the Supplementary Material). The results obtained from STRING were subsequently imported into Cytoscape software (version 3.9.1), a network biology visualization and analysis tool, which calculated the parameters for each node in the network graph and visualizes the molecular interactions (Shannon et al., 2003; Otasek et al., 2019). In addition, we verified the hub genes of these PPI networks using the MCODE plugin.

### 2.7 Molecular docking of phthalates to hub genes

Molecular docking was employed to investigate the potential binding interactions between the seven phthalates and the core targets. The PDB files of the key genes were downloaded from the Protein Data Bank website (https://www.rcsb.org/) (Zardecki et al., 2022), and the PDB of the key genes were removed from water and ligand using MOE, and then repaired with SPDBV. Ligand sdf files from PubChem were converted to PDB format using OpenBabel and Discovery Studio. The PDB of seven phthalates and key genes were processed with AutoDock 4.2 (Morris et al., 2009) and MGLTools 1.5.6 software. Hydrogenation of phthalates was saved as pdbqt format, and the pdb hydrogenation and charge of key genes were saved as pdbqt format. The processed phthalates and key genes were applied to Lamarckian genetic algorithm to complete the docking calculations, the number of docking was 50 times, and at the end of the run, a series of energy clusters were formed. The docking model that best-optimized energy was selected for result analysis, and the docking-binding conformation was visualized using PyMOL (Delano, 2002).

### 2.8 Expression analysis of key genes involved in the effect of phthalates on asthma and COPD diseases

In order to quantify the differential expression of key genes for the effects of seven phthalates on asthma and COPD disease compared to normal control tissues, epithelial tissue, and alveolar lavage fluid data for asthma were obtained using data from the GEO database [including normal controls as well as asthmatics (GSE67472: 62 cases and 43 healthy controls; GSE143303: 47 cases and 13 healthy controls)], with alveolar lavage fluid from asthmatics being subdivided into those with mild-to-medium-severe asthma (GSE74986: 12 healthy controls, 28 moderate asthma and 46 sever asthma) as well as COPD playing a role in epithelial cell gene expression data [mainly including airway epithelial cell tissues from orthostatic controls as well as COPD patients (GSE10066: 27 cases and 13 healthy controls, GSE11906: 33 cases and 56 healthy controls, GSE19407: 22 cases and 47 healthy controls)]. Violin plots of gene expression, representing the distribution of expression levels for each gene across different sample types, were constructed using the “ggplot2” package. The Wilcoxon rank-sum test was then applied to evaluate the expression level differences between these sample types, with the corresponding p-values calculated.

## 3 Result

### 3.1 Chemical information of 7 plasticizers

The chemical formulae, molecular weights, and SMILES structure details of the seven plasticizers are shown in Supplementary Table S1.

### 3.2 Target genes for 7 chemical compounds

After integrating the data, we found that DEHP targeted 123 genes, DBP targeted 105 genes, DEP targeted 114 genes, DIBP targeted 136 genes, DINP targeted 109 genes, DMP targeted 114 genes, and DOP targeted 173 genes. Some of these genes were shared among the seven phthalates, suggesting that they may have common health effect hazards.

### 3.3 Screening of asthma and COPD-related target genes

**FIGURE 2:**
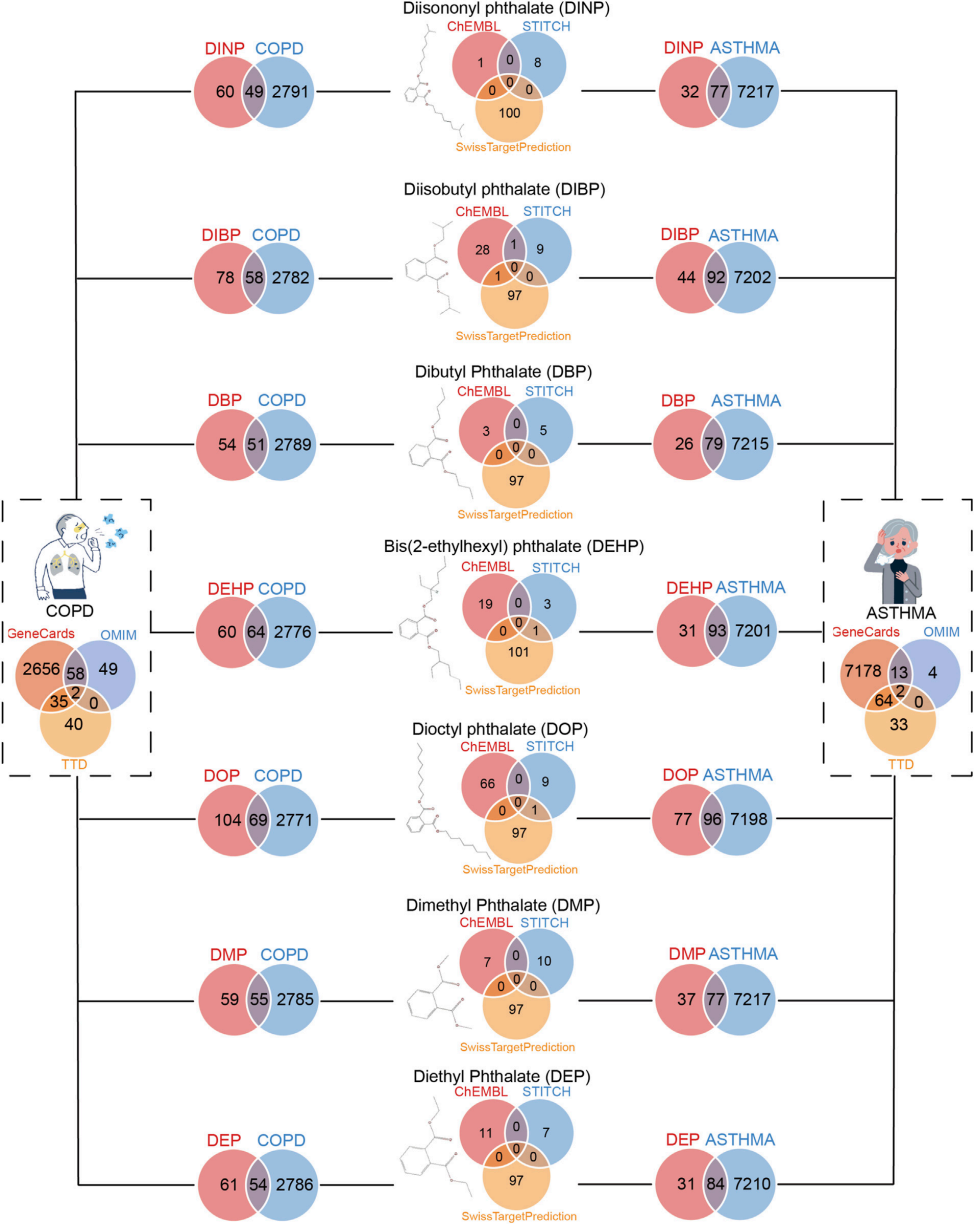
Screening for genes shared between benzoic acid compounds and respiratory diseases (asthma and COPD). On the left are genes that COPD patients shared with seven phthalates. On the right are genes that asthma patients shared with seven phthalates.

A total of 7,294 genes associated with asthma and 2,840 genes associated with COPD were identified for further analysis. As illustrated in Figure 2, the intersection of these asthma-related genes with the target genes of DEHP yielded 93 genes, with DBP yielding 79, DEP yielding 84, DIBP yielding 92, DINP yielding 77, with DMP yielding 77, DOP yielding 96. The intersection of COPD-related genes with the target genes of DEHP yielded 64 genes, 51 with DBP, with DEP, 58 with DIBP, 49 with DINP, 55 with DMP, and 69 with DOP. These genes represent potential targets for plasticizer-related asthma and COPD (Figure 2). We followed up with a functional enrichment analysis of these genes to explore the possible pathways by which different phthalates affect a disease. Further validation of the potential of these genes in serving as biomarkers for asthma and COPD is needed.

### 3.4 Functional enrichment analysis of target genes

We further analyzed the relationship of seven compounds with asthma, and COPD target genes to analyze and construct networks. A comprehensive Gene Ontology (GO) analysis was also conducted to elucidate the biological functions of the genes. Additionally, a Kyoto Encyclopedia of Genes and Genes (KEGG) analysis was performed to further identify the biological pathways in which the genes are involved. Our analysis generated GO entries for asthma and COPD on seven compounds categorized into biological process (BP), cellular component (CC), and molecular function (MF). The GO entries were ranked in accordance with the false discovery rate (FDR) values, with the top 10 entries exhibiting the lowest FDR values in the biological BP, CC, and MF categories selected for visual representation in enrichment analysis plots. Additionally, we conducted a KEGG analysis on potential targets to ascertain their involvement in particular signaling pathways. Furthermore, we generated statistically significant bubble plots, which visually represent the top 30 pathways in reverse order of FDR values**.** Among them, regarding asthma, we found in the GO analysis that the Integral component of synaptic membrane was common to all six compounds except DOP; five compounds except DINP and DOP acted on asthma through Endopeptidase activity; four compounds, DBP, DEHP, DIBP, and DMP, shared the Endolysosome; and three compounds, DBP, DEP, and DIBP, acted on asthma through Adenylate cyclase−modulating G protein−coupled receptor signaling pathway. 7 compounds were found in KEGG to act in asthma through Neuroactive ligand−receptor interaction; Apoptosis was common to 6 compounds except DINP; 5 compounds except DINP and DOP acted in asthma through Pathways of neurodegeneration − multiple diseases; Endocrine resistance was common to 4 compounds, DBP, DEHP, DEP, DMP; DBP, DEHP, and DEP, act in asthma through Measles. Regarding COPD, GO analysis indicated that Vacuolar lumen was common to all 7 compounds; all 6 compounds except DINP acted in COPD via Endolysosomes; and Ficolin-1-rich granule lumen was common to all 3 compounds, DBP, DEHP, and DIBP. KEGG analysis indicated that all seven compounds acted via Neuroactive ligand-receptor interaction in COPD; 6 compounds except DINP acted in COPD through Apoptosis, Prostate cancer, and AGE-RAGE signaling pathway in diabetic complications; EGFR tyrosine kinase inhibitor resistance was common to 4 compounds DEP, DINP, DMP, and DOP; and DEP, DMP, and DOP 3 compounds had PL3K-AKT signaling pathway. Subsequently, we selected the top 2 pathways in BP, CC, and MF and the top 6 pathways in KEGG rankings to generate functional enrichment analysis tables for asthma (Supplementary Table S2) and COPD (Supplementary Table S3), respectively, and refer to the Supplementary Material for complete results.

### 3.5 Construction of protein interaction networks and screening of core targets

**FIGURE 3:**
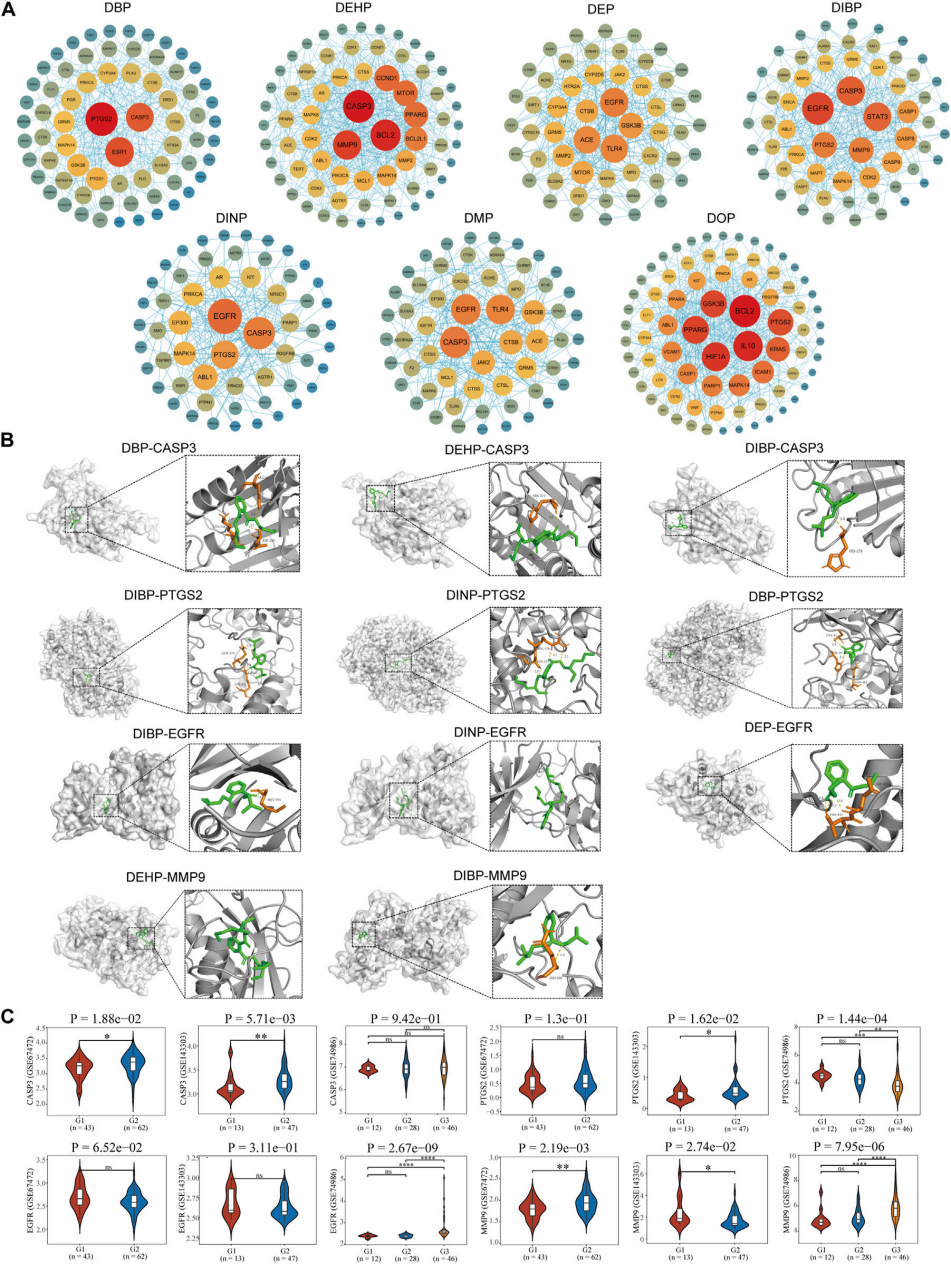
Identification of pivotal genes for the effects of phthalates on asthma. **(A)** Hub gene analysis of 7 phthalates affecting asthma. The closer the color to red and the larger the circle, the more important function of the gene. **(B)** The results of molecular docking of CASP3, PTGS2, EGFR and MMP9 with some phthalates are mainly shown. The orange represents the ligand, also known as the phthalate, the green represents the amino acid that binds to it, and the dotted line represents the hydrogen bond. **(C)** Differences in the expression of major asthma hub genes in normal and asthmatic lung epithelial tissue and in asthmatic alveolar lavage fluid were demonstrated. In GSE67472 and GSE143303 G1 represented normal lung epithelial tissue and G2 represented epithelial tissue from asthmatic patients. In GSE74986 G1 represented normal alveolar lavage fluid, G2 represented moderate asthma, and G3 represented alveolar lavage fluid from severe asthma. The Wilcoxon test was used to compare the two groups and the Kruskal–Wallis test was used to compare the three groups. *P* < 0.05 was considered statistically different.

**FIGURE 4:**
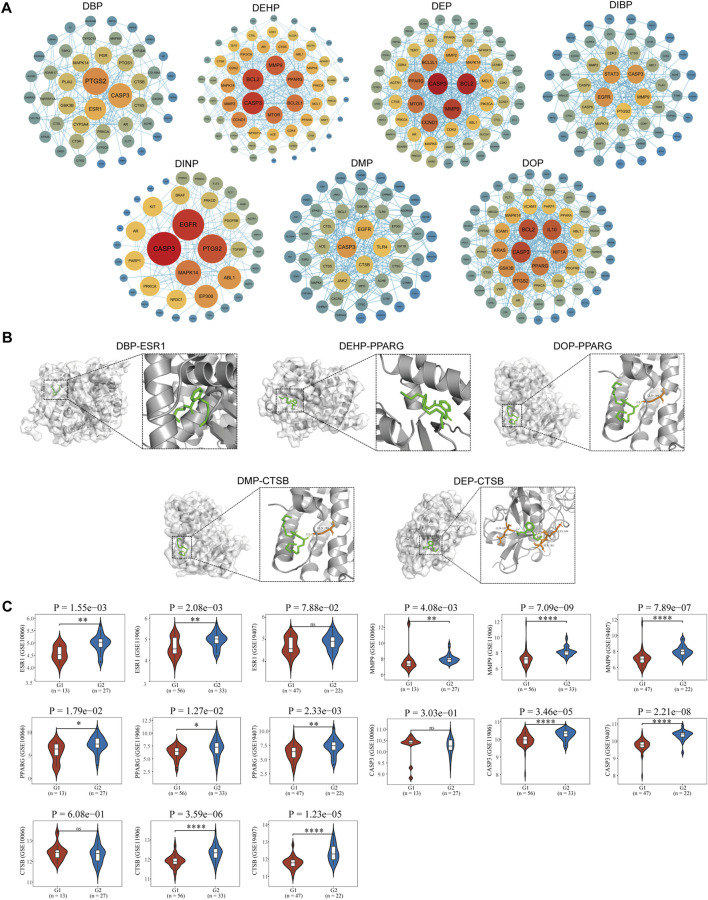
Identification of pivotal genes for the effects of phthalates on COPD. **(A)** Hub gene analysis of 7 phthalates affecting COPD. The closer the color to red and the larger the circle, the more important the function of the gene. **(B)** The molecular docking of ESR1 as well as PPARG and CTSB with DBP, DOP, DMP and CTSB were mainly demonstrated. (The duplicates have been shown in Figure 3.). **(C)** Expression of major pivotal genes in COPD lung epithelium and healthy lung epithelium. In all 3 datasets, G1 represents normal lung epithelium and G2 represents COPD lung epithelial tissue.

A protein-protein interaction network (PPI) was constructed using the STRING database, with nodes (representing genes) arranged in concentric circles based on their rank, which was determined by the composite centrality metric. Raw analytical data from the PPI were processed using Cytoscape software to create an optimized visualization of the protein-protein interaction network graph (Figures 3A, 4A). The size and color of the nodes reflect their respective degree values, with larger and more vibrant nodes indicating higher degrees. By analyzing the connectivity patterns and interactions within this network, provided insights into the interactions between seven different phthalates and COPD and asthma causative genes, respectively. Figure A represents the analysis of the hub genes for the interaction of the seven compounds and asthma, which revealed that the major hub genes for DBP were PTGS2, ESR1, and CASP3; for DEHP, CASP3, MMP9, BCL2, CCND1, MTOR, PPARG, and BCL2L2; for DEP, the major hub genes were EGFR, TLR4, ACE, and GSK3B; the major hub genes for DIBP were EGFR, CASP3, STAT3, MMP9, and PTGS2; the major hub genes for DINP were EGFR, CASP3, and PTGS2; the major hub genes for DMP were CASP3, EGFR, TLR4, CTSB, and JAK2; the major hub genes for DOP were BCL2, IL 10, HIF1A, PPARG, GSK3B, and PTGS2. We can find that some of the core genes are duplicated between different phthalates. Figure B represents the analysis of the hub genes for the action of the seven compounds and COPD, and it was found that the main hub genes for DBP were PTGS2, ESR1, and CASP3; the main hub genes for DEHP were CASP3, MMP9, BCL2, BCL2L1, MTOR, and PPARG; the main hub genes for DEP were CASP3, BCL2, MMP9, and CCND1; the major hub genes of DIBP are EGFR, CASP3, STAT3; the major hub genes of DINP are EGFR, CASP3, PTGS2, and MAPK14; the major hub genes of DMP are CASP3, EGFR, and TLR4; and the major hub genes of DOP are CASP3, BCL2, IL 10, and HIF1A. We can also find that the major genes in phthalates affecting COPD are also duplications between different types. And the main acting genes of phthalates affecting asthma and COPD are also duplications, suggesting that the compounds may have similar biological effects on the diseases.

### 3.6 Molecular docking analysis of seven phthalates with hub genes

In total, we performed molecular docking of seven phthalates and major hub genes. And the minimum number of dockings was set to 50. The binding energy is below −1.2 Kcal, which we consider to be a more stable binding state. There are a total of 38 docking results, with the left side of each figure showing the spatial binding structure of the protein and the specific compound, and the right side showing the detailed binding interactions and binding amino acids between the compound and the protein. We mainly presented the molecular docking results of the major hub genes for asthma, such as CASP3, PTGS2, EGFR, and MMP9 (Figure 3B), as well as the major hub genes for COPD, ESR1, PPARG, and CTSN (Figure 4B), with some of these phthalates **(**The detailed spatial structure of all dockings can be found in the Supplementary Material). We found that CASP3 binds to all seven phthalates with the lowest binding energy of −5.42 Kcal, with the same binding regions for DEHP and DOP, and the same binding regions for DEP and DMP in CASP3. Similarly, BCL2 binds to DEHP, DEP and DOP, with DEP being the most stable binding with a binding energy of −6.01 Kcal. and all three phthalates share the same binding site for BCL2. EGFR binds to all five phthalates except DBP and DEHP with binding energies below −1.2Kcal, with the same binding structural domains for DEP and DMP, and the same binding structural regions in EGFR for DIBP and DINP and DOP. The phthalates with the hub genes, binding profiles, binding energies and active amino acids are listed in Table 1.

**TABLE 1:** Summary of binding energies and bound amino acids for molecular docking.

PDB Id	Gene	PAEs	Binding energy/Kcal	Bound amino acid	Identical combination pocket
2J32	CASP3	DBP	−4.81	ARG93 ASP135 LYS138	DEHP-DOP DEP-DMP
		DEHP	−5.34	HIS277	
		DEP	−5.42	LYS38 TYR41	
		DIBP	−5.17	HIS278	
		DINP	−3.86	HIS277	
		DMP	−5.01	LYS38	
		DOP	−4.29	MET44	
6SBO	ESR1	DBP	−5.85	NONE	
5VAU	BCL2	DEHP	−4.98	NONE	DEHP-DEP-DOP
		DEP	−6.01	ARG127	
		DOP	−4.35	NONE	
8K5Y	MMP9	DEHP	−7.49	NONE	NONE
		DEP	−7.19	ARG249	
		DIBP	−7.29	GLN108	
2W96	CCND1	DEHP	−5.52	GLN188	
3FAP	MTOR	DEHP	−6.49	NONE	
5UG9	EGFR	DEP	−5.4	THR854 ASP855	DEP-DMP DIBP-DINP-DOP
		DIBP	−5.9	MET793	
		DINP	−5.9	NONE	
		DMP	−5.26	LYS745 THR854 ASP855	
		DOP	−5.02	GLY796	
1J1B	GSK3B	DEP	−6.63	ARG223 ARG723	DEP-DIBP
		DIBP	−7.92	ARG723 GLN765	
2Z62	TLR4	DEP	−5.52	ALA139 SER140 ASN143	NONE
		DMP	−5.28	ARG227	
6H5W	ACE	DEP	−5.95	LEU161 GLU162 CYS352	
8B4T	CTSB	DEP	−5.02	LYS184 SER185 GLU189	
		DMP	−5.02	HIS110 HIS111	
6QHD	STAT3	DIBP	−6.17	ARG325 GLN326	
5F19	PTGS2	DBP	−6.8	HIS39 CYS41 GLN461	
		DIBP	−7.51	GLN372 LYS532	
		DINP	−7.35	ASN375 ARG376	
3UGC	JAK2	DMP	−5.56	ASP994	
2ILK	IL10	DOP	−5.28	NONE	
4H6J	HIF1A	DOP	−4.7	LYS456	
8BF1	PPARG	DEHP	−6.23	NONE	
		DOP	−5.7	GLN286	
7JGW	BCL2L1	DEHP	−5.74	NONE	
2FST	MAPK14	DINP	−5.48	TRP197 ASN201	

### 3.7 Transcriptome analysis of key genes involved in the effects of phthalates on asthma and COPD disease

The acquired transcriptomic data were utilized for further expression analysis of the hub genes. A total of 6 GEO expression datasets were obtained, including respiratory epithelial cells from asthma patients and healthy control respiratory epithelial cells; and alveolar lavage gene expression data from normal control and asthma. The other three COPD data were all respiratory epithelial cell gene expression data from normal and COPD patients. In asthma we identified 16 core genes for phthalates, CASP3 was upregulated in two datasets in play epithelial cells, PTGS2 was upregulated only in GSE143303 but had a tendency to be upregulated in GSE67472, and CCND1 was downregulated in play epithelial cells; in combination with the alveolar lavage fluid data we found that TLR4, ESR1, PTGS2, IL10, IL10, TLR4, ESR2, and IL10 were not upregulated in play epithelial cells, and we found that TLR4 was upregulated in play epithelial cells. PTGS2, IL10, CTSB, JAK2, and MTOR were significantly downregulated in alveolar lavage fluid with increasing asthma severity, while MMP9, GSK3B, BCL2, EGFR, and CCND1 were upregulated. In COPD we finally identified a total of 13 hub genes with significant roles. In the three COPD datasets, the majority of gene expression trends exhibited consistency, with PPARG and MMP9 being upregulated in COPD airway epithelial cells. Additionally, ESR1, CTSB, and CASP3 were found to be upregulated in airway epithelial cells in two of the datasets, while BCL2 demonstrated downregulation; PTGS2, TLR4, IL10, and MTOR upregulated only in one of the datasets in play upregulated in epithelial cells and downregulated in EGFR. Specific statistical differences can be seen in the significance of difference markers of Figures 3C, 4C. A comparison of differences in all hub genes can be found in Supplementary Figure S1.

## 4 Discussion

The increasing incidence of asthma and chronic obstructive pulmonary disease (COPD) has heightened concerns about environmental factors, especially the impact of phthalate esters like di (2-ethylhexyl) phthalate (DEHP). Existing approaches have focused mainly on epidemiologic studies and toxicologic assessments, but gaps remain in understanding the molecular mechanisms by which phthalates affect respiratory health. This study used a network toxicology approach to elucidate the relationship between seven phthalates and respiratory diseases, specifically asthma and chronic obstructive pulmonary disease (COPD).

We performed hub targets screening for contaminants acting on diseases using chemoinformatic tools and molecular docking in computer-aided drug design (CADD) (Wadood et al., 2013). Computer-assisted drug design has been more helpful, for example, the application of molecular docking techniques in asthma can identify the target of action between the therapeutic drug and the disease (Arora et al., 2022b); in addition it can also help in subsequent pharmacological analyses (Arora et al., 2022c; Arora and Mesua ferrea, 2021). In our study, exposure to these phthalates was found to result in altered gene expression associated with inflammatory responses and airway hyperresponsiveness. The significance of this study lies in the identification of key pivotal genes that mediate the toxic effects of phthalates on respiratory health, providing new insights into the relevant molecular pathways. By combining chemical composition analysis with protein interaction networks, this study contributes to a more comprehensive understanding of how phthalates exacerbate respiratory diseases, thereby informing future prevention strategies and regulatory policies.

Asthma and COPD share similar pathophysiological processes in their development, primarily involving inflammation, oxidative stress, apoptosis, and changes in immune responses (Barnes, 2008). Prostaglandin-Endoperoxide Synthase 2 (PTGS2) plays a significant role in asthma and COPD, and its upregulation results in the production of mediators such as prostaglandins and further promotes airway inflammation, increasing symptoms of allergic asthma as well as exacerbations of COPD (Bao et al., 2022; Huang et al., 2024). Our study identified a strong association between four phthalates—DBP, DIBP, DINP, and DOP—and PTGS2. These phthalates were found to be upregulated in bronchial epithelial cells in both asthma and COPD. However, bronchoalveolar lavage analysis revealed a significant decrease in PTGS2 levels in moderate and severe asthma cases. The complexity of the role of inflammation in asthma is suggested and needs to be explored more fully. Matrix metalloproteinase 9 (MMP9), as a disease marker in COPD has been supported by evidence (Dimic-Janjic et al., 2023),Its upregulation has also been shown to be associated with the degree of asthma inflammation (Yan et al., 2024). In asthma DEHP, DIBP were strongly associated with MMP9,and in COPD DEHP, DEP, DIBP were associated with MMP9. The transcriptome found significantly elevated MMP9 in bronchial epithelium in asthma and COPD, and alveolar lavage fluid in asthma also confirmed significant upregulation of MMP9 in moderate and severe asthma. The findings highlight the significant role of DEHP and DIBP in influencing both asthma and COPD. Among the cysteine proteases, caspase-3 plays a crucial role in regulating apoptosis, and the pathogenesis and pathophysiology of both asthma and COPD are closely linked to apoptotic processes (Pandey et al., 2017). In asthma, all phthalates except DOP exert their toxic effects via CASP3; in COPD, all phthalates exert their toxic effects via CASP3, showing that phthalates share a common mechanism of toxicity in both asthma and COPD. CASP3 is markedly overexpressed in bronchial epithelial cells in patients with asthma and COPD, but is not significantly altered in alveolar lavage fluid of asthma patients. B-cell lymphoma 2 (BCL2) is mainly an anti-apoptotic protein and is involved in the biological process of apoptosis in asthma and COPD. B-cell lymphoma 2 (BCL2) is primarily an anti-apoptotic protein, and apoptotic biological processes are involved in asthma and COPD (Jin et al., 2019; Liu et al., 2023). Our analysis also revealed that BCL2 was downregulated in bronchial epithelium in asthma and COPD, although no statistically significant difference was observed between asthma patients and normal controls. The results of our analysis revealed that DEHP, DOP affected asthma through BCL2, whereas DEHP and DEP and DOP affected COPD through BCL2. Interestingly, in alveolar lavage fluid, BCL2 was elevated in asthmatics with increasing asthma severity, which may be related to inhibition of apoptosis by BCL2, increasing the risk of severe asthma cancer. Estrogen Receptor 1 (ESR1) is an important intranuclear receptor involved in the regulation of cellular responses to estrogen, with implications for immune responses and inflammatory processes. ESR was downregulated in bronchial epithelial tissues of asthma (with no statistically significant difference compared to normal controls), and decreased in alveolar lavage fluid with increasing disease severity, suggesting that downregulation of ESR1 may be associated with the anti-inflammatory effects may be related to the weakening of the anti-inflammatory effects of estrogen. However, it is upregulated in bronchial epithelial tissues in COPD, which may suggest that the main role of ESR1 in COPD is to play a role in remodeling rather than inflammation. Our findings that DBP exerts its toxic effects through ESR1 in both asthma and COPD are of value for further detailed exploration. CTSB, on the other hand, may have a similar role to ESR1, which is downregulated in alveolar lavage fluid in severe asthma as well as upregulated in bronchial epithelial cells in COPD. Only DMP acts through CTSB in both diseases. CTSB shows different modulation patterns in COPD and asthma, reflecting the different pathophysiological processes of the two diseases. In COPD, the upregulation of CTSB may be related to long-term chronic inflammation and airway remodeling, whereas the downregulation of CTSB in asthma may be related to specific allergic response mechanisms and protective regulation. Mammalian target of rapamycin (MTOR) is a key signaling protein that plays a crucial role in various physiological processes, including cell growth, proliferation, metabolism, and autophagy. Its associated signaling pathway is related to T cell differentiation, and chronic inflammation and plays a role in remodeling (Arora et al., 2024). DEHP may act through MTOR in asthma and COPD and participate in asthma inflammation and airway remodeling in COPD. Transcriptome analysis confirmed that upregulation of MTOR was also found in bronchial epithelial cells in asthma and COPD. There are also some genes whose expression is more interesting and needs to be explored in more depth. Toll-like receptor 4 (TLR4), whose upregulation is associated with increased inflammation in asthma (Yang et al., 2024). However, we found that TLR4 was significantly decreased in alveolar lavage fluid in severe asthma, which may be due to the fact that in the inflammatory milieu of severe asthma, some inhibitory factors may be produced, which can downregulate the expression of TLR4. These factors may include some anti-inflammatory cytokines or other regulatory factors. Epidermal Growth Factor Receptor (EGFR), was significantly upregulated in severe asthma but significantly decreased in bronchial epithelial cells in COPD. We suggest that airway inflammation is particularly prominent in patients with severe asthma and that the upregulation of EGFR may be a response to inflammatory mediators such as cytokines. The activation of EGFR stimulates the proliferation of airway smooth muscle cells, collagen deposition, and the progression of airway remodeling. This may also be a mechanism for the transition from asthma to COPD.

As well as the above hub genes that are common to both asthma and COPD, there are also hub genes that are unique to each condition. Cyclin D1 (CCND1), a unique hub gene for DEHP action in asthma, is associated with the rest of the cell cycle, and its upregulation would further increase potentially exacerbate airway wall thickening and airway remodelling. We found that CCND1 upregulation in alveolar lavage fluid was positively associated with asthma severity. However, the expression of CCND1 is markedly reduced in the bronchial epithelial cells of individuals with asthma in comparison to those without the condition. One possible mechanism is that asthmatics are in a state of prolonged oxidative stress, which may block the cell cycle, but CCND1 in alveolar lavage fluid is synthesised by a different cell and therefore the total expression is higher. Glycogen synthase kinase 3β (GSK3B), whose elevation has been shown to be associated with asthma (Noori et al., 2019). DSK3B is a unique hub gene for DOP action in asthma, and there is no literature linking the two that is valuable to study in depth. Our analyses also found that GSK3B was upregulated in bronchoalveolar lavage fluid in asthma and positively correlated with asthma severity. Janus kinase 2 (JAK2), a unique hub gene for DMP, is an important regulator associated with asthma inflammation (Zhang et al., 2024). Although its upregulation was associated with increased inflammation in asthma, our analysis revealed that JAK2 was significantly decreased in both asthmatic bronchial epithelium and alveolar lavage fluid in severe asthma, which is inconsistent with the current study and suggests that the mechanism of action is complex.

Limitations of this study stem mainly from the reliance on a methodology that, while useful, may not entirely capture the complexity of biological systems *in vivo*. In addition, the selection of phthalates analyzed was not comprehensive; future studies should consider a broader range of compounds to cover potential synergistic effects. In addition, variations in individual susceptibility to respiratory diseases may not have been adequately accounted for, suggesting that further longitudinal studies are required to validate the findings.

## 5 Conclusion

This investigation clarified the detrimental effects of seven phthalates on respiratory diseases, especially asthma and chronic obstructive pulmonary disease (COPD), and established the epidemiological relevance of these conditions. Using network toxicology, the study identified key genes affected by phthalates, highlighting their involvement in protein interaction networks, thereby emphasizing disease mechanisms. The analysis of key gene expression further validated the harmful impact of these chemicals on respiratory health. This study has greatly contributed to the understanding of environmental toxins in respiratory diseases by emphasizing the need for regulatory review of phthalates and advocating for strengthened public health policies to reduce exposures and protect susceptible populations.

## Data Availability

The original contributions presented in the study are included in the article/Supplementary Material, further inquiries can be directed to the corresponding author.

## References

[B1] GBD 2015 Chronic Respiratory Disease Collaborators (2017). Global, regional, and national deaths, prevalence, disability-adjusted life years, and years lived with disability for chronic obstructive pulmonary disease and asthma, 1990-2015: a systematic analysis for the Global Burden of Disease Study 2015. Lancet Respir. Med. 5 (9), 691–706. 10.1016/S2213-2600(17)30293-X 28822787 PMC5573769

[B2] Adgent M. A. Carroll K. N. Hazlehurst M. F. Loftus C. T. Szpiro A. A. Karr C. J. (2020). A combined cohort analysis of prenatal exposure to phthalate mixtures and childhood asthma. Environ. Int. 143, 105970. 10.1016/j.envint.2020.105970 32763629 PMC7708520

[B3] Arbuckle T. E. Davis K. Marro L. Fisher M. Legrand M. LeBlanc A. (2014). Phthalate and bisphenol A exposure among pregnant women in Canada--results from the MIREC study. Environ. Int. 68, 55–65. 10.1016/j.envint.2014.02.010 24709781

[B4] Arora P. Ansari S. H. Nainwal L. M. (2022c). Clerodendrum serratum extract attenuates production of inflammatory mediators inovalbumin-induced asthma in rats. Turkish J. Chem. 46 (2), 330–341. 10.3906/kim-2107-22 PMC10734715 38143476

[B5] Arora P. Athari S. S. Nainwal L. M. (2022a). Piperine attenuates production of inflammatory biomarkers, oxidative stress and neutrophils in lungs of cigarette smoke-exposed experimental mice. Food Biosci. 49, 101909. 10.1016/j.fbio.2022.101909

[B6] Arora P. Mesua ferrea L. (2021). Calophyllaceae exerts therapeutic effects in allergic asthma by modulating cytokines production in asthmatic rats. Turkish J. Bot. 45 (SI-2), 820–832. 10.3906/bot-2111-22

[B7] Arora P. Mohan Nainwal L. (2024). “Chapter Seven - association between T-helper cell-mediated immune response and airway remodeling in allergic asthma,” in Allergic asthma: immunopathogenesis. Editors Athari S. S. Nasab E. M. (Academic Press), 167–179.

[B8] Arora P. Nainwal L. M. Gupta G. (2022b). Orally administered solasodine, a steroidal glycoalkaloid, suppresses ovalbumin-induced exaggerated Th2-immune response in rat model of bronchial asthma. Chemico-Biological Interact., 366. 10.1016/j.cbi.2022.110138 36084726

[B9] Bao C. Liu C. Liu Q. Hua L. Hu J. Li Z. (2022). Liproxstatin-1 alleviates LPS/IL-13-induced bronchial epithelial cell injury and neutrophilic asthma in mice by inhibiting ferroptosis. Int. Immunopharmacol. 109, 108770. 10.1016/j.intimp.2022.108770 35483233

[B10] Barnes P. J. (2008). Immunology of asthma and chronic obstructive pulmonary disease. Nat. Rev. Immunol. 8 (3), 183–192. 10.1038/nri2254 18274560

[B11] Benjamin S. Pradeep S. Josh M. S. Kumar S. Masai E. (2015). A monograph on the remediation of hazardous phthalates. J. Hazard Mater 298, 58–72. 10.1016/j.jhazmat.2015.05.004 26004054

[B12] Berger K. Coker E. Rauch S. Eskenazi B. Balmes J. Kogut K. (2020). Prenatal phthalate, paraben, and phenol exposure and childhood allergic and respiratory outcomes: evaluating exposure to chemical mixtures. Sci. Total Environ. 725, 138418. 10.1016/j.scitotenv.2020.138418 32302842 PMC7255953

[B13] Brassea-Pérez E. Hernández-Camacho C. J. Labrada-Martagón V. Vázquez-Medina J. P. Gaxiola-Robles R. Zenteno-Savín T. (2022). Oxidative stress induced by phthalates in mammals: state of the art and potential biomarkers. Environ. Res. 206, 112636. 10.1016/j.envres.2021.112636 34973198

[B14] Delano W. L. (2002). The PyMOL molecular graphics system.

[B15] Dimic-Janjic S. Hoda M. A. Milenkovic B. Kotur-Stevuljevic J. Stjepanovic M. Gompelmann D. (2023). The usefulness of MMP-9, TIMP-1 and MMP-9/TIMP-1 ratio for diagnosis and assessment of COPD severity. Eur. J. Med. Res. 28 (1), 127. 10.1186/s40001-023-01094-7 36935521 PMC10026402

[B16] Duh T. H. Yang C. J. Lee C. H. Ko Y. C. (2023). A study of the relationship between phthalate exposure and the occurrence of adult asthma in taiwan. Molecules 28 (13), 5230. 10.3390/molecules28135230 37446892 PMC10343260

[B17] Halbert R. J. Natoli J. L. Gano A. Badamgarav E. Buist A. S. Mannino D. M. (2006). Global burden of COPD: systematic review and meta-analysis. Eur. Respir. J. 28 (3), 523–532. 10.1183/09031936.06.00124605 16611654

[B18] He N. Zhang J. Liu M. Yin L. (2024). Elucidating the mechanism of plasticizers inducing breast cancer through network toxicology and molecular docking analysis. Ecotoxicol. Environ. Saf. 284, 116866. 10.1016/j.ecoenv.2024.116866 39178760

[B19] Huang S. (2024). A novel strategy for the study on molecular mechanism of prostate injury induced by 4,4'-sulfonyldiphenol based on network toxicology analysis. J. Appl. Toxicol. 44 (1), 28–40. 10.1002/jat.4506 37340727

[B20] Huang Y. Niu Y. Wang X. Li X. He Y. Liu X. (2024). Identification of novel biomarkers related to neutrophilic inflammation in COPD. Front. Immunol. 15, 1410158. 10.3389/fimmu.2024.1410158 38873611 PMC11169582

[B21] Hulin M. Simoni M. Viegi G. Annesi-Maesano I. (2012). Respiratory health and indoor air pollutants based on quantitative exposure assessments. Eur. Respir. J. 40 (4), 1033–1045. 10.1183/09031936.00159011 22790916

[B22] Jin A. Bao R. Roth M. Liu L. Yang X. Tang X. (2019). microRNA-23a contributes to asthma by targeting BCL2 in airway epithelial cells and CXCL12 in fibroblasts. J. Cell Physiol. 234 (11), 21153–21165. 10.1002/jcp.28718 31020662

[B23] Jurewicz J. Hanke W. (2011). Exposure to phthalates: reproductive outcome and children health. A review of epidemiological studies. Int. J. Occup. Med. Environ. Health 24 (2), 115–141. 10.2478/s13382-011-0022-2 21594692

[B24] Kasper-Sonnenberg M. Koch H. M. Wittsiepe J. Brüning T. Wilhelm M. (2014). Phthalate metabolites and bisphenol A in urines from German school-aged children: results of the Duisburg birth cohort and Bochum cohort studies. Int. J. Hyg. Environ. Health 217 (8), 830–838. 10.1016/j.ijheh.2014.06.001 24986699

[B25] Lee G. H. Kim J. H. Kim S. Lee S. Lim D. H. (2020). Effects of indoor air purifiers on children with asthma. Yonsei Med. J. 61 (4), 310–316. 10.3349/ymj.2020.61.4.310 32233173 PMC7105409

[B26] Leynaert B. Le Moual N. Neukirch C. Siroux V. Varraso R. (2019). Environmental risk factors for asthma developement. Presse Med. 48 (3 Pt 1), 262–273. 10.1016/j.lpm.2019.02.022 30910274

[B27] Li S. (2016). Exploring traditional Chinese medicine by a novel therapeutic concept of network target. Chin. J. Integr. Med. 22 (9), 647–652. 10.1007/s11655-016-2499-9 27145941

[B28] Liu X. Ma Y. Luo L. Zeng Z. Zong D. Chen Y. (2023). Taxifolin ameliorates cigarette smoke-induced chronic obstructive pulmonary disease via inhibiting inflammation and apoptosis. Int. Immunopharmacol. 115, 109577. 10.1016/j.intimp.2022.109577 36584569

[B29] Liye L. Hui Z. Fuchun H. Hua L. (2024). Research progress of airway inflammation in asthma: a bibliometric analysis. Med. Baltim. 103 (29), e38842. 10.1097/MD.0000000000038842 PMC11398817 39029036

[B30] Marie C. Vendittelli F. Sauvant-Rochat M. P. (2015). Obstetrical outcomes and biomarkers to assess exposure to phthalates: a review. Environ. Int. 83, 116–136. 10.1016/j.envint.2015.06.003 26118330

[B31] Morris G. M. Huey R. Lindstrom W. Sanner M. F. Belew R. K. Goodsell D. S. (2009). AutoDock4 and AutoDockTools4: automated docking with selective receptor flexibility. J. Comput. Chem. 30 (16), 2785–2791. 10.1002/jcc.21256 19399780 PMC2760638

[B32] Nabi S. A. Ramzan F. Lone M. S. (2022). Synthesis, crystallographic study, molecular docking, ADMET, DFT and biological evaluation of new series of aurone derivatives as anti-leishmanial agents. J. Mol. Struct., 1256.

[B33] Nainwal L. M. Parida P. Das A. Bairy P. S. (2014). Design and docking of novel series of hybrid xanthones as anti-cancer agent to target human DNA topoisomerase 2-alpha. Bangladesh J. Pharmacol. 9 (2). 10.3329/bjp.v9i2.18180

[B34] Nainwal L. M. Shaququzzaman M. Akhter M. Husain A. Parvez S. Khan F. (2020). Synthesis, ADMET prediction and reverse screening study of 3,4,5-trimethoxy phenyl ring pendant sulfur‐containing cyanopyrimidine derivatives as promising apoptosis inducing anticancer agents. Bioorg. Chem. 104, 104282. 10.1016/j.bioorg.2020.104282 33010624

[B35] Nainwal L. M. Shaququzzaman M. Akhter M. Husain A. Parvez S. Tasneem S. (2022). Synthesis, and reverse screening of 6-(3,4,5-trimethoxyphenyl)pyrimidine-5-carbonitrile derivatives as anticancer agents: Part-II. J. Heterocycl. Chem. 59 (4), 771–788. 10.1002/jhet.4421

[B36] Noori M. S. Bhatt P. M. Courreges M. C. Ghazanfari D. Cuckler C. Orac C. M. (2019). Identification of a novel selective and potent inhibitor of glycogen synthase kinase-3. Am. J. Physiol. Cell Physiol. 317 (6), C1289-C1303–c1303. 10.1152/ajpcell.00061.2019 31553649 PMC6962522

[B37] Odebeatu C. C. Taylor T. Fleming L. E. N J. O. (2019). Phthalates and asthma in children and adults: US NHANES 2007-2012. Environ. Sci. Pollut. Res. Int. 26 (27), 28256–28269. 10.1007/s11356-019-06003-2 31368075 PMC6791917

[B38] Otasek D. Morris J. H. Bouças J. Pico A. R. Demchak B. (2019). Cytoscape Automation: empowering workflow-based network analysis. Genome Biol. 20 (1), 185. 10.1186/s13059-019-1758-4 31477170 PMC6717989

[B39] Pandey K. C. De S. Mishra P. K. (2017). Role of proteases in chronic obstructive pulmonary disease. Front. Pharmacol. 8, 512. 10.3389/fphar.2017.00512 28848433 PMC5550664

[B40] Polakoff P. L. Lapp N. L. Reger R. (1975). Polyvinyl chloride pyrolysis products. A potential cause for respiratory impairment. Arch. Environ. Health 30 (6), 269–271. 10.1080/00039896.1975.10666697 1137432

[B41] Quirós-Alcalá L. Belz D. C. Woo H. Lorizio W. Putcha N. Koehler K. (2023). A cross sectional pilot study to assess the role of phthalates on respiratory morbidity among patients with chronic obstructive pulmonary disease. Environ. Res. 225, 115622. 10.1016/j.envres.2023.115622 36894111 PMC10580394

[B42] Shannon P. Markiel A. Ozier O. Baliga N. S. Wang J. T. Ramage D. (2003). Cytoscape: a software environment for integrated models of biomolecular interaction networks. Genome Res. 13 (11), 2498–2504. 10.1101/gr.1239303 14597658 PMC403769

[B43] Tao W. Xu X. Wang X. Li B. Wang Y. Li Y. (2013). Network pharmacology-based prediction of the active ingredients and potential targets of Chinese herbal Radix Curcumae formula for application to cardiovascular disease. J. Ethnopharmacol. 145 (1), 1–10. 10.1016/j.jep.2012.09.051 23142198

[B44] Wadood A. Ahmed N. Shah L. Ahmad A. Hassan H. Shams S. (2013). In-silico drug design: an approach which revolutionarised the drug discovery process. Drug Des. Deliv. 1. 10.13172/2054-4057-1-1-1119

[B45] Yan Q. Zhang X. Xie Y. Yang J. Liu C. Zhang M. (2024). Bronchial epithelial transcriptomics and experimental validation reveal asthma severity-related neutrophilc signatures and potential treatments. Commun. Biol. 7 (1), 181. 10.1038/s42003-024-05837-y 38351296 PMC10864370

[B46] Yang Z. Li X. Wei L. Bao L. Hu H. Liu L. (2024). Involucrasin B suppresses airway inflammation in obese asthma by inhibiting the TLR4-NF-κB-NLRP3 pathway. Phytomedicine 132, 155850. 10.1016/j.phymed.2024.155850 39029138

[B47] Zardecki C. Dutta S. Goodsell D. S. Lowe R. Voigt M. Burley S. K. (2022). PDB-101: educational resources supporting molecular explorations through biology and medicine. Protein Sci. 31 (1), 129–140. 10.1002/pro.4200 34601771 PMC8740840

[B48] Zhang Z. He Y. Liu H. Wu T. Li R. (2024). NLRP3 regulates ferroptosis via the JAK2/STAT3 pathway in asthma inflammation: insights from *in vivo* and *in vitro* studies. Int. Immunopharmacol. 143 (Pt 2), 113416. 10.1016/j.intimp.2024.113416 39426227

[B49] Zhao A. Wang L. Pang X. Liu F. (2022). Phthalates in skin wipes: distribution, sources, and exposure via dermal absorption. Environ. Res. 204 (Pt B), 112041. 10.1016/j.envres.2021.112041 34529968

