# Supplementary material for: The impact of phthalates on asthma and chronic obstructive pulmonary disease: a comprehensive analysis based on network toxicology and molecular docking
Source: Front Pharmacol. 2025 Mar 14;16:1566965. doi: 10.3389/fphar.2025.1566965 (PMC11949918; doi:10.3389/fphar.2025.1566965)
Supplement: Supplementary file 1 [file Table1.docx]

| Type | Databases | link |
| --- | --- | --- |
| Compound | ChEMBL | <https://www.ebi.ac.uk/chembl/> |
|  | STITCH | <http://stitch.embl.de/> |
|  | SwissTargetPrediction | <http://www.swisstargetprediction.ch/> |
| Disease | GeneCards | <https://www.genecards.org/> |
|  | OMIM | <https://omim.org/> |
|  | TTD | <http://db.idrblab.net/ttd/> |
| Netwotk | PPI | <https://string-db.org/> |

**Table S1. Major databases and links to sources of compounds and disease genes**
