# Supplementary material for: The impact of phthalates on asthma and chronic obstructive pulmonary disease: a comprehensive analysis based on network toxicology and molecular docking
Source: Front Pharmacol. 2025 Mar 14;16:1566965. doi: 10.3389/fphar.2025.1566965 (PMC11949918; doi:10.3389/fphar.2025.1566965)
Supplement: Supplementary file 2 [file Table2.docx]

| Compound | ID (GO) | ONTOLOGY | Description | p.adjust | ID (KEGG) | Description | p.adjust |
| --- | --- | --- | --- | --- | --- | --- | --- |
|  | GO:0007188 | BP | adenylate cyclase-modulating G protein-coupled receptor signaling pathway | 6.06E-13 | hsa04080 | Neuroactive ligand-receptor interaction | 1.93E-13 |
|  | GO:0003018 |  | vascular process in circulatory system | 1.26E-12 | hsa04726 | Serotonergic synapse | 2.49E-07 |
|  | GO:0099055 | CC | integral component of postsynaptic membrane | 1.92E-06 | hsa04723 | Retrograde endocannabinoid signaling | 2.48E-06 |
|  | GO:0097060 |  | synaptic membrane | 1.92E-06 | hsa04668 | TNF signaling pathway | 2.48E-06 |
|  | GO:0030594 | MF | neurotransmitter receptor activity | 4.96E-10 | hsa04917 | Prolactin signaling pathway | 3.54E-06 |
|  | GO:0008227 |  | G protein-coupled amine receptor activity | 7.36E-09 | hsa05120 | Epithelial cell signaling in Helicobacter pylori infection | 3.54E-06 |
| DEHP | GO:0042391 | BP | regulation of membrane potential | 8.55E-10 | hsa04933 | AGE-RAGE signaling pathway in diabetic complications | 4.82E-08 |
|  | GO:0032496 |  | response to lipopolysaccharide | 1.13E-09 | hsa04210 | Apoptosis | 6.38E-08 |
|  | GO:0000307 | CC | cyclin-dependent protein kinase holoenzyme complex | 8.39E-07 | hsa04080 | Neuroactive ligand-receptor interaction | 1.34E-07 |
|  | GO:0031256 |  | leading edge membrane | 1.32E-06 | hsa04115 | p53 signaling pathway | 1.34E-07 |
|  | GO:0004175 | MF | endopeptidase activity | 9.24E-06 | hsa04218 | Cellular senescence | 1.52E-07 |
|  | GO:0004114 |  | 3',5'-cyclic-nucleotide phosphodiesterase activity | 9.24E-06 | hsa05415 | Diabetic cardiomyopathy | 3.68E-07 |
| DEP | GO:0023061 | BP | signal release | 1.21E-13 | hsa04080 | Neuroactive ligand-receptor interaction | 6.06E-16 |
|  | GO:0001505 |  | regulation of neurotransmitter levels | 1.21E-13 | hsa04210 | Apoptosis | 2.28E-05 |
|  | GO:0099699 | CC | integral component of synaptic membrane | 3.99E-09 | hsa04020 | Calcium signaling pathway | 2.28E-05 |
|  | GO:0097060 |  | synaptic membrane | 3.99E-09 | hsa04024 | cAMP signaling pathway | 3.46E-05 |
|  | GO:0030594 | MF | neurotransmitter receptor activity | 9.51E-13 | hsa05224 | Breast cancer | 2.59E-04 |
|  | GO:0008227 |  | G protein-coupled amine receptor activity | 3.98E-12 | hsa05210 | Colorectal cancer | 4.02E-04 |
| DIBP | GO:1903522 | BP | regulation of blood circulation | 4.58E-10 | hsa04080 | Neuroactive ligand-receptor interaction | 6.18E-09 |
|  | GO:0007188 |  | adenylate cyclase-modulating G protein-coupled receptor signaling pathway | 1.53E-09 | hsa04210 | Apoptosis | 7.00E-07 |
|  | GO:0045211 | CC | postsynaptic membrane | 6.11E-09 | hsa04723 | Retrograde endocannabinoid signaling | 1.32E-06 |
|  | GO:0097060 |  | synaptic membrane | 2.55E-08 | hsa05022 | Pathways of neurodegeneration - multiple diseases | 7.23E-06 |
|  | GO:0004175 | MF | endopeptidase activity | 8.01E-12 | hsa05032 | Morphine addiction | 9.37E-06 |
|  | GO:0030594 |  | neurotransmitter receptor activity | 4.62E-11 | hsa05010 | Alzheimer disease | 3.32E-05 |
| DINP | GO:0071900 | BP | regulation of protein serine/threonine kinase activity | 6.46E-11 | hsa04080 | Neuroactive ligand-receptor interaction | 1.69E-06 |
|  | GO:0043434 |  | response to peptide hormone | 8.34E-11 | hsa04020 | Calcium signaling pathway | 2.39E-06 |
|  | GO:0032589 | CC | neuron projection membrane | 4.95E-04 | hsa04010 | MAPK signaling pathway | 8.67E-05 |
|  | GO:0031256 |  | leading edge membrane | 4.95E-04 | hsa04072 | Phospholipase D signaling pathway | 1.96E-03 |
|  | GO:0042562 | MF | hormone binding | 9.78E-10 | hsa04928 | Parathyroid hormone synthesis, secretion and action | 2.25E-03 |
|  | GO:0004713 |  | protein tyrosine kinase activity | 1.07E-06 | hsa04015 | Rap1 signaling pathway | 2.63E-03 |
| DMP | GO:0001505 | BP | regulation of neurotransmitter levels | 2.74E-11 | hsa04080 | Neuroactive ligand-receptor interaction | 1.77E-09 |
|  | GO:0050804 |  | modulation of chemical synaptic transmission | 1.41E-09 | hsa04210 | Apoptosis | 9.54E-08 |
|  | GO:0045211 | CC | postsynaptic membrane | 4.61E-10 | hsa04725 | Cholinergic synapse | 3.06E-04 |
|  | GO:0097060 |  | synaptic membrane | 1.68E-09 | hsa04142 | Lysosome | 6.21E-04 |
|  | GO:0030594 | MF | neurotransmitter receptor activity | 3.67E-10 | hsa04024 | cAMP signaling pathway | 6.21E-04 |
|  | GO:0098960 |  | postsynaptic neurotransmitter receptor activity | 3.94E-09 | hsa05162 | Measles | 6.21E-04 |
| DOP | GO:0043434 | BP | response to peptide hormone | 9.25E-11 | hsa05417 | Lipid and atherosclerosis | 1.09E-07 |
|  | GO:0090257 |  | regulation of muscle system process | 3.55E-10 | hsa04933 | AGE-RAGE signaling pathway in diabetic complications | 6.33E-07 |
|  | GO:0036019 | CC | endolysosome | 6.16E-05 | hsa04210 | Apoptosis | 9.22E-07 |
|  | GO:0045121 |  | membrane raft | 3.28E-04 | hsa04080 | Neuroactive ligand-receptor interaction | 1.55E-06 |
|  | GO:0008528 | MF | G protein-coupled peptide receptor activity | 1.52E-06 | hsa04148 | Efferocytosis | 2.49E-05 |
|  | GO:0001653 |  | peptide receptor activity | 1.52E-06 | hsa04370 | VEGF signaling pathway | 8.96E-05 |

**Table S2. Analysis of GO and KEGG for seven intersecting genes for phthalates and asthma (FDR<0.05).**
