# Supplementary material for: The impact of phthalates on asthma and chronic obstructive pulmonary disease: a comprehensive analysis based on network toxicology and molecular docking
Source: Front Pharmacol. 2025 Mar 14;16:1566965. doi: 10.3389/fphar.2025.1566965 (PMC11949918; doi:10.3389/fphar.2025.1566965)
Supplement: Supplementary file 3 [file Table3.docx]

| Compound | ID(GO) | ONTOLOGY | Description | p.adjust | ID (KEGG) | Description | p.adjust |
| --- | --- | --- | --- | --- | --- | --- | --- |
| DBP | GO:0042445 | BP | hormone metabolic process | 7.33E-07 | hsa04080 | Neuroactive ligand-receptor interaction | 9.48E-06 |
|  | GO:0031281 | BP | positive regulation of cyclase activity | 7.33E-07 | hsa04726 | Serotonergic synapse | 1.60E-05 |
|  | GO:0036019 | CC | endolysosome | 1.25E-04 | hsa04210 | Apoptosis | 3.89E-05 |
|  | GO:0031904 | CC | endosome lumen | 1.91E-04 | hsa04020 | Calcium signaling pathway | 4.07E-05 |
|  | GO:0004175 | MF | endopeptidase activity | 1.47E-05 | hsa04917 | Prolactin signaling pathway | 6.91E-05 |
|  | GO:0020037 | MF | heme binding | 1.47E-05 | hsa05120 | Epithelial cell signaling in Helicobacter pylori infection | 6.91E-05 |
| DEHP | GO:0032496 | BP | response to lipopolysaccharide | 4.04E-12 | hsa04933 | AGE-RAGE signaling pathway in diabetic complications | 1.56E-08 |
|  | GO:0002237 | BP | response to molecule of bacterial origin | 5.32E-12 | hsa04210 | Apoptosis | 1.56E-08 |
|  | GO:0036019 | CC | endolysosome | 6.39E-06 | hsa05415 | Diabetic cardiomyopathy | 1.17E-06 |
|  | GO:0031904 | CC | endosome lumen | 1.32E-05 | hsa01522 | Endocrine resistance | 1.46E-06 |
|  | GO:0004175 | MF | endopeptidase activity | 2.57E-06 | hsa05166 | Human T-cell leukemia virus 1 infection | 1.73E-06 |
|  | GO:0004674 | MF | protein serine/threonine kinase activity | 3.59E-05 | hsa04115 | p53 signaling pathway | 1.73E-06 |
| DEP | GO:0009410 | BP | response to xenobiotic stimulus | 2.08E-07 | hsa04080 | Neuroactive ligand-receptor interaction | 3.09E-08 |
|  | GO:0007187 | BP | G protein-coupled receptor signaling pathway, coupled to cyclic nucleotide second messenger | 2.08E-07 | hsa04210 | Apoptosis | 4.88E-07 |
|  | GO:0031904 | CC | endosome lumen | 1.30E-07 | hsa04142 | Lysosome | 5.81E-05 |
|  | GO:0036019 | CC | endolysosome | 1.15E-06 | hsa01522 | Endocrine resistance | 7.06E-05 |
|  | GO:0004175 | MF | endopeptidase activity | 1.00E-09 | hsa04020 | Calcium signaling pathway | 7.06E-05 |
|  | GO:0017171 | MF | serine hydrolase activity | 1.16E-09 | hsa04024 | cAMP signaling pathway | 1.89E-04 |
| DIBP | GO:0062197 | BP | cellular response to chemical stress | 4.54E-07 | hsa04210 | Apoptosis | 2.08E-06 |
|  | GO:0032496 | BP | response to lipopolysaccharide | 6.61E-07 | hsa05161 | Hepatitis B | 5.91E-06 |
|  | GO:0036019 | CC | endolysosome | 3.78E-06 | hsa04933 | AGE-RAGE signaling pathway in diabetic complications | 1.32E-05 |
|  | GO:0062023 | CC | collagen-containing extracellular matrix | 2.83E-04 | hsa05205 | Proteoglycans in cancer | 2.42E-05 |
|  | GO:0004175 | MF | endopeptidase activity | 3.88E-08 | hsa05163 | Human cytomegalovirus infection | 4.97E-05 |
|  | GO:0004197 | MF | cysteine-type endopeptidase activity | 3.88E-08 | hsa04912 | GnRH signaling pathway | 5.78E-05 |
| DINP | GO:0043410 | BP | positive regulation of MAPK cascade | 1.44E-09 | hsa04010 | MAPK signaling pathway | 1.06E-06 |
|  | GO:0043406 | BP | positive regulation of MAP kinase activity | 1.51E-09 | hsa04015 | Rap1 signaling pathway | 1.23E-05 |
|  | GO:0031256 | CC | leading edge membrane | 7.74E-04 | hsa04928 | Parathyroid hormone synthesis, secretion and action | 1.23E-05 |
|  | GO:0032589 | CC | neuron projection membrane | 1.12E-03 | hsa04020 | Calcium signaling pathway | 4.89E-05 |
|  | GO:0004713 | MF | protein tyrosine kinase activity | 2.63E-08 | hsa04520 | Adherens junction | 4.47E-04 |
|  | GO:0019199 | MF | transmembrane receptor protein kinase activity | 2.21E-07 | hsa04933 | AGE-RAGE signaling pathway in diabetic complications | 5.99E-04 |
| DMP | GO:0098926 | BP | postsynaptic signal transduction | 2.39E-08 | hsa04210 | Apoptosis | 6.44E-08 |
|  | GO:1902074 | BP | response to salt | 2.39E-08 | hsa04142 | Lysosome | 8.27E-05 |
|  | GO:0031904 | CC | endosome lumen | 1.63E-07 | hsa04080 | Neuroactive ligand-receptor interaction | 8.27E-05 |
|  | GO:0036019 | CC | endolysosome | 1.28E-06 | hsa04725 | Cholinergic synapse | 2.39E-04 |
|  | GO:0004197 | MF | cysteine-type endopeptidase activity | 7.89E-10 | hsa05152 | Tuberculosis | 3.97E-04 |
|  | GO:0004175 | MF | endopeptidase activity | 7.89E-10 | hsa05162 | Measles | 5.56E-04 |
| DOP | GO:0043434 | BP | response to peptide hormone | 5.70E-09 | hsa04933 | AGE-RAGE signaling pathway in diabetic complications | 6.60E-07 |
|  | GO:0090257 | BP | regulation of muscle system process | 5.70E-09 | hsa05417 | Lipid and atherosclerosis | 3.97E-06 |
|  | GO:0036019 | CC | endolysosome | 8.34E-06 | hsa04210 | Apoptosis | 3.97E-06 |
|  | GO:0009897 | CC | external side of plasma membrane | 8.34E-06 | hsa04722 | Neurotrophin signaling pathway | 1.47E-04 |
|  | GO:0001653 | MF | peptide receptor activity | 1.29E-04 | hsa04015 | Rap1 signaling pathway | 1.47E-04 |
|  | GO:0004197 | MF | cysteine-type endopeptidase activity | 1.29E-04 | hsa04370 | VEGF signaling pathway | 1.85E-04 |

**Table S3. Analysis of GO and KEGG for seven intersecting genes for phthalates and COPD (FDR<0.05).**
